# Supplementary material for: Diversity and evolution of the endosymbionts of Bemisia tabaci in China
Source: PeerJ. 2018 Aug 29;6:e5516. doi: 10.7717/peerj.5516 (PMC6119459; doi:10.7717/peerj.5516)
Supplement: Supplemental Information 1 [file peerj-06-5516-s001.docx]

**Table S1 Sampling information of *B. tabaci* used in this study, and the infection rates of *P. aleyrodidarum*, *Arsenophous* and *Cardinium* within populations.**

| **Location** | **Host plant** | **Species** | **ntot** | **Port.**  **Prev.** | **Arsen.** **Prev.** | **Cardi. Prev.** |
| --- | --- | --- | --- | --- | --- | --- |
| Changchun,Jiling | *Euphorbia pulcherrima* | MEAM1 | 10 | 100% | – | – |
| Tianjin | *Solanum melongena* | MEAM1 | 10 | 100% | – | – |
| Tianjin | *Gossypium spp* | MEAM1 | 10 | 100% | – | – |
| Tianjin | *Cucumis sativus* | MEAM1 | 10 | 100% | – | – |
| Beijing | *Abutilon theophrasti* | MEAM1 | 10 | 100% | – | – |
| Zhenzhou,Henan | *Capsicum annuum* | MEAM1 | 10 | 100% | – | – |
| Zhenzhou,Henan | *Cucumis sativus* | MEAM1 | 10 | 100% | – | – |
| Xuzhou,Jiangsu | *Solanum melongena* | MEAM1 | 10 | 100% | – | – |
| Xuzhou,Jiangsu | *Luffa cylindrica* | MEAM1 | 10 | 100% | – | – |
| Xuzhou,Jiangsu | *Cucurbita moschata* | MEAM1 | 10 | 100% | – | – |
| Xuzhou,Jiangsu | *Solanum melongena* | MEAM1 | 10 | 100% | – | – |
| Xuzhou,Jiangsu | *Cucurbita moschata* | MEAM1 | 10 | 100% | – | – |
| Huaian,Jiangsu | *Vigna unguiculata* | MEAM1 | 10 | 100% | – | – |
| Huaian,Jiangsu | *Solanum melongena* | MEAM1 | 10 | 100% | – | – |
| Taigu,Shanxi | *Zinnia elegans* | MEAM1 | 10 | 100% | – | – |
| Taigu,Shanxi | *Nicotiana tabacum* | MEAM1 | 10 | 100% | – | – |
| Taigu,Shanxi | *Pharbifis nil* | MEAM1 | 10 | 100% | – | – |
| Taigu,Shanxi | *Gossypium spp* | MEAM1 | 10 | 100% | – | – |
| Zhaotong,Yunnan | *Brassica rapa* | MEAM1 | 10 | 100% | – | – |
| Zhaotong,Yunnan | *Solanum melongena* | MEAM1 | 10 | 100% | – | – |
| Panzhihua,Sichuan | *Cucumis sativus* | MEAM1 | 10 | 100% | – | 20% |
| Panzhihua,Sichuan | *Solanum melongena* | MEAM1 | 10 | 100% | – | 40% |
| Zunyi,Guizhou | *Solanum melongena* | MEAM1 | 10 | 100% | – | – |
| Rizhao,Shandong | *Vigna unguiculata* | MEAM1 | 10 | 100% | – | – |
| Rizhao,Shandong | *Cucumis sativus* | MEAM1 | 10 | 100% | – | – |
| Tai'an,Shandong | *Nicotiana tabacum* | MEAM1 | 10 | 100% | – | – |
| Tai'an,Shandong | *Lactuca sativa* | MEAM1 | 10 | 100% | – | – |
| Tai'an,Shandong | *Brassica oleracea* | MEAM1 | 10 | 100% | – | – |
| Tai'an,Shandong | *Raphanus sativus* | MEAM1 | 10 | 100% | – | – |
| ZhanZhou.Hainan | *Solanum melongena* | MEAM1 | 10 | 100% | – | – |
| Sanya,Hainan | *Citrullus lanatus* | MEAM1 | 10 | 100% | – | – |
| Qionghai,Hainan | *Glycine max* | MEAM1 | 10 | 100% | – | – |
| Baisha,Hainan | *Solanum melongena* | MEAM1 | 10 | 100% | – | – |
| Dening,Fujian | *Cucumis sativus* | MEAM1 | 10 | 100% | – | – |
| Dening,Fujian | *Cucumis sativus* | MEAM1 | 10 | 100% | – | – |
| Fuzhou,Fujian | *Ipomoea batatas* | MEAM1 | 10 | 100% | – | – |
| Fuzhou,Fujian | *Gossypium spp* | MEAM1 | 10 | 100% | – | – |
| Nanping,Fujian | *Glycine max* | MEAM1 | 10 | 100% | – | – |
| Longyan,Fujian | *Nicotiana tabacum* | MEAM1 | 10 | 100% | – | – |
| Foshan,Guangdong | *Cucumis sativus* | MEAM1 | 10 | 100% | – | – |
| Foshan,Guangdong | *Solanum melongena* | MEAM1 | 10 | 100% | – | – |
| Foshan,Guangdong | *Brassica rapa* | MEAM1 | 10 | 100% | – | – |
| Zhongshan,Guangdong | *Brassica rapa* | MEAM1 | 10 | 100% | – | 30% |
| Zhongshan,Guangdong | *Solanum melongena* | MEAM1 | 10 | 100% | – | 10% |
| Huizhou,Guangdong | *Cucumis sativus* | MEAM1 | 10 | 100% | – | – |
| Huizhou,Guangdong | *Solanum melongena* | MEAM1 | 10 | 100% | – | – |
| Huizhou,Guangdong | *Vigna unguiculata* | MEAM1 | 10 | 100% | – | – |
| Dongwan,Guangdong | *Solanum melongena* | MEAM1 | 10 | 100% | – | – |
| Shantou,Guangdong | *Vigna unguiculata* | MEAM1 | 10 | 100% | – | – |
| Shantou,Guangdong | *Cucurbita pepo* | MEAM1 | 10 | 100% | – | – |
| Shantou,Guangdong | *Brassica rapa* | MEAM1 | 10 | 100% | – | – |
| Shantou,Guangdong | *Cucumis sativus* | MEAM1 | 10 | 100% | – | – |
| Shantou,Guangdong | *Solanum melongena* | MEAM1 | 10 | 100% | – | – |
| Wenyuan,Guangdong | *Glycine max* | MEAM1 | 10 | 100% | – | – |
| Wenyuan,Guangdong | *Solanum melongena* | MEAM1 | 10 | 100% | – | – |
|  |  | **MEAM1** | **550** |  |  |  |
| Shenyang,Liaoning | *Euphorbia pulcherrima* | MED | 10 | 100% | – | 30% |
| Beijing | *Pharbifis nil* | MED | 10 | 100% | – | – |
| Beijing | *Gossypium spp* | MED | 10 | 100% | – | 70% |
| Beijing | *Solanum melongena* | MED | 10 | 100% | – | 100% |
| Hebi,Henan | *Gossypium spp* | MED | 10 | 100% | – | 10% |
| Zhenzhou,Henan | *Solanum melongena* | MED | 10 | 100% | 70% | – |
| Zhenzhou,Henan | *Lycopersicon esculentum* | MED | 10 | 100% | – | 80% |
| Zhenzhou,Henan | *Nicotiana tabacum* | MED | 10 | 100% | – | – |
| Zhenzhou,Henan | *Gossypium spp* | MED | 10 | 100% | 30% | 100% |
| Hebei | *Solanum melongena* | MED | 10 | 100% | 80% | 80% |
| Hebei | *Capsicum annuum* | MED | 10 | 100% | 30% | – |
| Hebei | *Cucumis sativus* | MED | 10 | 100% | 20% | – |
| Hebei | *Lycopersicon esculentum* | MED | 10 | 100% | 40% | – |
| Hebei | *Gossypium spp* | MED | 10 | 100% | 100% | – |
| Shanghai | *Ipomoea batatas* | MED | 10 | 100% | – | – |
| Shanghai | *Nicotiana tabacum* | MED | 10 | 100% | – | 100% |
| Lianyungang,Jiangsu | *Cucumis sativus* | MED | 10 | 100% | – | – |
| Huaian,Jiangsu | *Vigna unguiculata* | MED | 10 | 100% | – | – |
| Nanjing,Jiangsu | *Nicotiana tabacum* | MED | 10 | 100% | – | 30% |
| Yancheng,Jiangsu | *Cucumis sativus* | MED | 10 | 100% | – | 40% |
| Yangzhou,Jiangsu | *Vigna unguiculata* | MED | 10 | 100% | – | – |
| Xi'an,Shanxi | *Capsicum annuum* | MED | 10 | 100% | – | – |
| Xi'an,Shanxi | *Nicotiana tabacum* | MED | 10 | 100% | – | – |
| Ganzhong,Shanxi | *Euphorbia pulcherrima* | MED | 10 | 100% | – | 100% |
| Changsha,Hunan | *Nicotiana tabacum* | MED | 10 | 100% | – | 100% |
| Changsha,Hunan | *Cucumis sativus* | MED | 10 | 100% | – | 100% |
| Wuhan,Hubei | *Nicotiana tabacum* | MED | 10 | 100% | – | – |
| Taigu,Shanxi | *Cucumis sativus* | MED | 10 | 100% | – | – |
| Linfen,Shanxi | *Solanum melongena* | MED | 10 | 100% | 30% | – |
| Linfen,Shanxi | *Cucumis sativus* | MED | 10 | 100% | – | – |
| Anshun,Guizhou | *Cucumis sativus* | MED | 10 | 100% | – | 60% |
| Bengbu,Anhui | *Humulus japonicus* | MED | 10 | 100% | – | – |
| Bengbu,Anhui | *Gossypium spp* | MED | 10 | 100% | – | – |
| Hexian,Anhui | *Solanum melongena* | MED | 10 | 100% | – | – |
| Qingdao,Shandong | *Euphorbia pulcherrima* | MED | 10 | 100% | – | 20% |
| Laiwu,Shandong | *Solanum melongena* | MED | 10 | 100% | 10% | – |
| Dezhou,Shandong | *Nicotiana tabacum* | MED | 10 | 100% | – | – |
| Dezhou,Shandong | *Benincasa hispida* | MED | 10 | 100% | – | – |
| Dezhou,Shandong | *Brassica rapa* | MED | 10 | 100% | – | – |
| Dezhou,Shandong | *Gossypium spp* | MED | 10 | 100% | – | – |
| Dezhou,Shandong | *Pharbifis nil* | MED | 10 | 100% | – | – |
| Dezhou,Shandong | *Helianthus tuberosus* | MED | 10 | 100% | – | – |
| Heze,Shandong | *Solanum melongena* | MED | 10 | 100% | – | – |
| Heze,Shandong | *Cucumis sativus* | MED | 10 | 100% | 100% | – |
| Liangshan,Shandong | *Vigna unguiculata* | MED | 10 | 100% | – | – |
| Liangshan,Shandong | *Nicotiana tabacum* | MED | 10 | 100% | – | – |
| Ji'ning,Shandong | *Nicotiana tabacum* | MED | 10 | 100% | – | – |
| Huishan,Shandong | *Gossypium spp* | MED | 10 | 100% | – | – |
| Huishan,Shandong | *Capsicum annuum* | MED | 10 | 100% | – | – |
| Huishan,Shandong | *Nicotiana tabacum* | MED | 10 | 100% | 20% | – |
| Huishan,Shandong | *Solanum melongena* | MED | 10 | 100% | 30% | – |
| Jiaxiang,Shandong | *Capsicum annuum* | MED | 10 | 100% | – | – |
| Jiaxiang,Shandong | *Solanum melongena* | MED | 10 | 100% | 30% | – |
| Jiaxiang,Shandong | *Nicotiana tabacum* | MED | 10 | 100% | 60% | – |
| Zaozhuang,Shandong | *Gossypium spp* | MED | 10 | 100% | – | – |
| Tengzhou,Shandong | *Humulus japonicus* | MED | 10 | 100% | – | 100% |
| Dongfang,Hainan | *Ricinus communis* | MED | 10 | 100% | 40% | – |
| Baoting,Hainan | *Solanum melongena* | MED | 10 | 100% | – | 100% |
| Ninghai,Zhejiang | *Cucumis sativus* | MED | 10 | 100% | – | – |
| Shenzhen,Guangdong | *Argyreia seguinii* | MED | 10 | 100% | – | – |
|  |  | **MED** | **600** |  |  |  |
| Meizhou,Guangdong | *Ipomoea batatas* | Asia Ⅱ1 | 10 | 100% | 60% | 100% |
| Meizhou,Guangdong | *Lactuca sativa* | Asia Ⅱ1 | 10 | 100% | 70% | 70% |
| Qingyuan,Guangdong | *Brassica campestris* | Asia Ⅱ1 | 10 | 100% | 60% | – |
| Qingyuan,Guangdong | *Arachis hypogaea* | Asia Ⅱ1 | 10 | 100% | 60% | 80% |
| Qingyuan,Guangdong | *Vigna unguiculata* | Asia Ⅱ1 | 10 | 100% | – | – |
| Qingyuan,Guangdong | *Glycine max* | Asia Ⅱ1 | 10 | 100% | 50% | – |
| Qingyuan,Guangdong | *Ipomoea batatas* | Asia Ⅱ1 | 10 | 100% | 90% | – |
|  |  | **Asia Ⅱ1** | **70** |  |  |  |
| Wuhan,Hubei | *Gossypium spp* | Asia Ⅱ3 | 10 | 100% | – | – |
| Nanchang,Jiangxi | *Solanum melongena* | Asia Ⅱ3 | 10 | 100% | – | – |
| Jiuhuashan,Anhui | *Gossypium spp* | Asia Ⅱ3 | 10 | 100% | 50% | 30% |
| Linghai,Zhejiang | *Glycine max* | Asia Ⅱ3 | 10 | 100% | 100% | 40% |
| Wenzhou,Zhejiang | *Ipomoea batatas* | Asia Ⅱ3 | 10 | 100% | 100% | 100% |
| Shenzhen,Guangdong | *Hibiscus schizopetalus* | Asia Ⅱ3 | 10 | 100% | 100% | 100% |
| Shenzhen,Guangdong | *Argyreia seguinii* | Asia Ⅱ3 | 10 | 100% | 90% | 100% |
| Shenzhen,Guangdong | *Humulus japonicus* | Asia Ⅱ3 | 10 | 100% | – | 100% |
| Shenzhen,Guangdong | *Vigna unguiculata* | Asia Ⅱ3 | 10 | 100% | 80% | 100% |
| Heyuan,Guangdong | *Ipomoea batatas* | Asia Ⅱ3 | 10 | 100% | 40% | – |
| Heyuan,Guangdong | *Lactuca sativa* | Asia Ⅱ3 | 10 | 100% | – | – |
| Heyuan,Guangdong | *Brassica rapa* | Asia Ⅱ3 | 10 | 100% | 90% | – |
| Heyuan,Guangdong | *Arachis hypogaea* | Asia Ⅱ3 | 10 | 100% | – | – |
| Qingyuan,Guangdong | *Lactuca sativa* | Asia Ⅱ3 | 10 | 100% | 60% | – |
|  |  | **Asia Ⅱ3** | **140** |  |  |  |
| Yongchuan,Chongqin | *Vigna radiata* | China 1 | 10 | 100% | 60% | – |
| Yongchuan,Chongqin | *Solanum melongena* | China 1 | 10 | 100% | 100% | – |
| Yongchuan,Chongqin | *Vigna unguiculata* | China 1 | 10 | 100% | 100% | – |
| Guang'an,Sichuan | *Solanum melongena* | China 1 | 10 | 100% | 40% | – |
| Ganzhou,Jiangxi | *Glycine max* | China 1 | 10 | 100% | 100% | – |
| Liu'an,Anhui | *Nicotiana tabacum* | China 1 | 10 | 100% | 30% | – |
| Liu'an,Anhui | *Cucumis sativus* | China 1 | 10 | 100% | 60% | – |
| Liu'an,Anhui | *Solanum melongena* | China 1 | 10 | 100% | 30% | – |
| Liu'an,Anhui | *Vigna unguiculata* | China 1 | 10 | 100% | 30% | – |
| Wuyi,Fujian | *Glycine max* | China 1 | 10 | 100% | 100% | – |
| Yiwu,Zhejiang | *Humulus japonicus* | China 1 | 10 | 100% | – | – |
| Shengzhou,Zhejiang | *Solanum melongena* | China 1 | 10 | 100% | – | – |
| Xiaoshan,Zhejiang | *Humulus japonicus* | China 1 | 10 | 100% | – | – |
| Meizhou,Guangdong | *Argyreia seguinii* | China 1 | 10 | 100% | 100% | – |
| Shaoguan,Guangdong | *Lactuca sativa* | China 1 | 10 | 100% | 80% | – |
|  |  | **China 1** | **150** |  |  |  |
|  |  |  | **1510** |  |  |  |

ntot: number of individuals screened for test. *Port.* Prev.: *P. aleyrodidarum* prevalence. *Arsen.* Prev.: *Arsenophous* prevalence. *Cardi.* Prev.: *Cardinium* prevalence. –: negative for test.
